# ASAP: a platform for gene functional analysis in *Angelica sinensis*

**DOI:** 10.1186/s12864-024-09971-z

**Published:** 2024-01-23

**Authors:** Silan Wu, Lingling Da, Qiaoqiao Xiao, Qi Pan, Jinqiang Zhang, Jiaotong Yang

**Affiliations:** 1 Resource Institute for Chinese and Ethnic Materia MedicaGuizhou University of Traditional Chinese Medicine, Guizhou, 550025 China; 2 College of Life Science, Northwest Normal University, Lanzhou, China

**Keywords:** *Angelica sinensis*, Functional annotation, Analysis tools, Platform

## Abstract

**Background:**

*Angelica sinensis* (Danggui), a renowned medicinal orchid, has gained significant recognition for its therapeutic effects in treating a wide range of ailments. Genome information serves as a valuable resource, enabling researchers to gain a deeper understanding of gene function. In recent times, the availability of chromosome-level genomes for *A. sinensis* has opened up vast opportunities for exploring gene functionality. Integrating multiomics data can allow researchers to unravel the intricate mechanisms underlying gene function in *A. sinensis* and further enhance our knowledge of its medicinal properties.

**Results:**

In this study, we utilized genomic and transcriptomic data to construct a coexpression network for *A. sinensis*. To annotate genes, we aligned them with sequences from various databases, such as the NR, TAIR, trEMBL, UniProt, and SwissProt databases. For GO and KEGG annotations, we employed InterProScan and GhostKOALA software. Additionally, gene families were predicted using iTAK, HMMER, OrholoFinder, and KEGG annotation. To facilitate gene functional analysis in *A. sinensis*, we developed a comprehensive platform that integrates genomic and transcriptomic data with processed functional annotations. The platform includes several tools, such as BLAST, GSEA, Heatmap, JBrowse, and Sequence Extraction. This integrated resource and approach will enable researchers to explore the functional aspects of genes in *A. sinensis* more effectively.

**Conclusion:**

We developed a platform, named ASAP, to facilitate gene functional analysis in *A. sinensis*. ASAP (www.gzybioinformatics.cn/ASAP) offers a comprehensive collection of genome data, transcriptome resources, and analysis tools. This platform serves as a valuable resource for researchers conducting gene functional research in their projects, providing them with the necessary data and tools to enhance their studies.

**Supplementary Information:**

The online version contains supplementary material available at 10.1186/s12864-024-09971-z.

## Background

*Angelica sinensis* is an herbal plant used in traditional Chinese medicine and belongs to the Apiaceae subfamily [1]. Its historical use in culture and medicine traces back approximately 2000 years ago to the Han Dynasty of China, as documented in Shennong's *Classic of Materia Medica* [2]. *A. sinensis* has been utilized not only as a health food and medicinal herb in Asian countries but also as a component of dietary supplements for women's health in Europe and North America [3]. *A. sinensis* contains a diverse array of coumarins, which serve as important compounds for both plant growth and as sources of antiviral drugs [4]. Recent pharmacological studies have provided compelling evidence for the remarkable therapeutic potential of *A. sinensis*. Indeed, it has been found to possess significant antitumor and antiarrhythmic properties while also bolstering the immune system and effectively neutralizing harmful free radicals through its potent antioxidant activity [5, 6].

As an increasing number of plant genomes are being described, numerous platforms and databases for studying plant gene functions are being continuously released. Examples include RoseAP [7], GelFAP [8], TAIR [9], and LjaFGD [10]. Recently, the genome of *A. sinensis* was also explored at the chromosomal level [1]. Moreover, a plethora of transcriptome information has been obtained to investigate various aspects of *A. sinensis*, such as its flowering, stress resistance, and medicinal properties. For instance, Peng et al. [11] conducted a transcriptome analysis to explore the impact of ultraviolet B (UV-B) radiation on two different variants of *A. sinensis*, and Li et al. [12] investigated the molecular mechanisms of flowering by comparing bolted and unbolted *A. sinensis*. Additionally, Feng et al. [13] utilized transcriptome and metabolite profile analyses to identify 108 potential candidate isoforms associated with phthalide accumulation.

These databases and transcriptome data serve as valuable resources for gene functional analysis and the utilization of existing data. In this study, we constructed a gene function analysis platform for *A. sinensis* based on the chromosome-level genome that provides a reference for users to carry out studies on gene function and active component synthesis pathways.

## Materials and methods

### Data resources

The genome sequences, CDSs, and protein sequences of *A. sinensis* were retrieved from the genome sequencing data obtained by Han et al. [1] and deposited in the cyVerse platform. The NR protein database was sourced from the sequence library maintained by the National Center for Biotechnology Information (NCBI) at https://ftp.ncbi.nlm.nih.gov/blast/db/. TrEMBL, SwissProt, and UniProt sequences were obtained from the UniProt database [14]; TAIR protein sequences were obtained through the download interface provided by the TAIR website [9]. Transcriptome data were collected from the NCBI Sequence Read Archive (SRA) database. KEGG annotation information was acquired from the KEGG database, and GO annotation information was obtained from agriGO v2 [15]. Plant EAR motif-containing protein sequences were sourced from the PlantEAR database [16], carbohydrate-active enzymes (CAZy) protein sequences originated from the CAZy database [17], and transport protein (TP) sequences were derived from TransportDB [18].

### Functional annotation

The protein sequences of *A. sinensis* were aligned with protein sequences from public databases, including the NR, UniProt, SwissProt, and TAIR databases, using Diamond Blast [19], and the best match from these databases was selected as the annotation information result. KEGG annotations were predicted using the GhostKOALA website [20], and the predicted KEGG numbers were used to retrieve annotation information from the KEGG database [21]. GO annotations were assessed using InterProScan software [22] to obtain GO numbers, and the corresponding annotation information was downloaded from agriGO v2.0 [15] based on GO numbers. Pfam domain information was predicted using InterProScan software [22].

### Coexpression network construction

First, we used HISAT2 software [23] to map the downloaded transcriptome data to the reference genome of *A. sinensis* and obtain BAM files. We subsequently used SAMtools [24] to sort the BAM files. Next, we used Stringtie software [25] to obtain the expression values of each transcriptome sample and construct an expression matrix. We calculated the correlation between gene expression for every pair of genes using the PCC algorithm. After that, we ranked the gene correlations using the MR algorithm. Finally, we evaluated the network using receiver operating characteristic (ROC) curves and selected an appropriate threshold to construct a coexpression network. The formula is as follows:

$$PCC=\frac{\sum (X-\overline{X })(Y-\overline{Y })}{\sqrt{{\sum }_{i=1}^{n}{({X}_{i}-\overline{X })}^{2}}\sqrt{{\sum }_{i=1}^{n}{({Y}_{i}-\overline{Y })}^{2}}}$$

$$MR\left(AB\right)=\sqrt{Rank(AB)\times Rank(BA)}$$

In the given formulas, 'n' represents the total number of samples in the RNA-seq data, while 'X' and 'Y' represent the TPM values. The term 'Rank' refers to the order of the PCC values, where 'AB' signifies the ranking of gene A among all the genes with gene B, and 'BA' indicates the reverse ranking.

Moreover, we assessed the network's reliability and established specific threshold values for both the PCC and MR metrics. As part of the analysis, we identified Gene Ontology (GO) terms related to biological process entries, specifically focusing on those with gene counts ranging from 4 to 20; these genes were designated as prior gene sets. Additionally, we selected genes coexpressed with the genes under the defined threshold to form other gene sets. By comparing areas under the ROC curve (AUCs) at various thresholds, we determined the PCCs and MRs that yielded the maximum AUC, representing the optimal intersection between the two types of gene sets. Furthermore, we retained the three genes with the highest PCC values for each gene.

### Protein‒protein interaction (PPI) network

To construct the PPI network for *A. sinensis*, we employed OrthoFinder software [26] to predict orthologous relationships between Arabidopsis and *A. sinensis*. Subsequently, we mapped the PPI network from Arabidopsis to *A. sinensis.*

### Gene family identification

Initially, we utilized OrthoFinder [26] to predict the orthologous relationships between proteins of *Arabidopsis* and *A. sinensis*. Subsequently, we identified proteins containing CAZy, TP, and EAR motifs based on these orthologous relationships. To identify and classify transcription factors and protein kinases in *A. sinensis*, we utilized iTAK software (Plant Transcription Factor & Protein Kinase Identifier and Classifier) [27], which is available at http://bioinfo.bti.cornell.edu/cgi-bin/itak/index.cgi. Moreover, by using a hidden Markov model obtained from iUUCD 2.0 (http://uucd.biocuckoo.org/) [28], we identified ubiquitin families in *A. sinensis*. Annotation of KEGG pathways for the entire genome was accomplished using GhostKOALA [20]. Additionally, functional annotation of the CYP450 genes was performed based on KEGG annotations. Except for the EAR motif-containing proteins classified as other, all predicted gene families were identified using the Pfam domain.

### Construction of ASAP

Construction of the platform was based on the Linux, Apache, MySQL, and PHP (LAMP) technical stack. By importing all relevant results and data analysis, including gene structure annotation, gene functional annotation, coexpression network, PPI network, and gene family classification, a MySQL database was created. To facilitate data presentation and analysis, dynamic websites were developed using the HTML, PHP, JavaScript, and CSS languages.

### Toolkit for gene function analysis

Gene set enrichment analysis (GSEA) [29] was performed with the platform following previous methods [8, 30, 31]. For BLAST analysis, we utilized ViroBlast [32]. JBrowse software [33], which was developed by Buels et al., was integrated into our platform to showcase omics information. Additionally, we implemented a sequence extraction tool using a Perl script and introduced a heatmap analysis tool based on Highchart Javascript.

## Platform contents

### Gene functional annotation

**Fig. 1:**
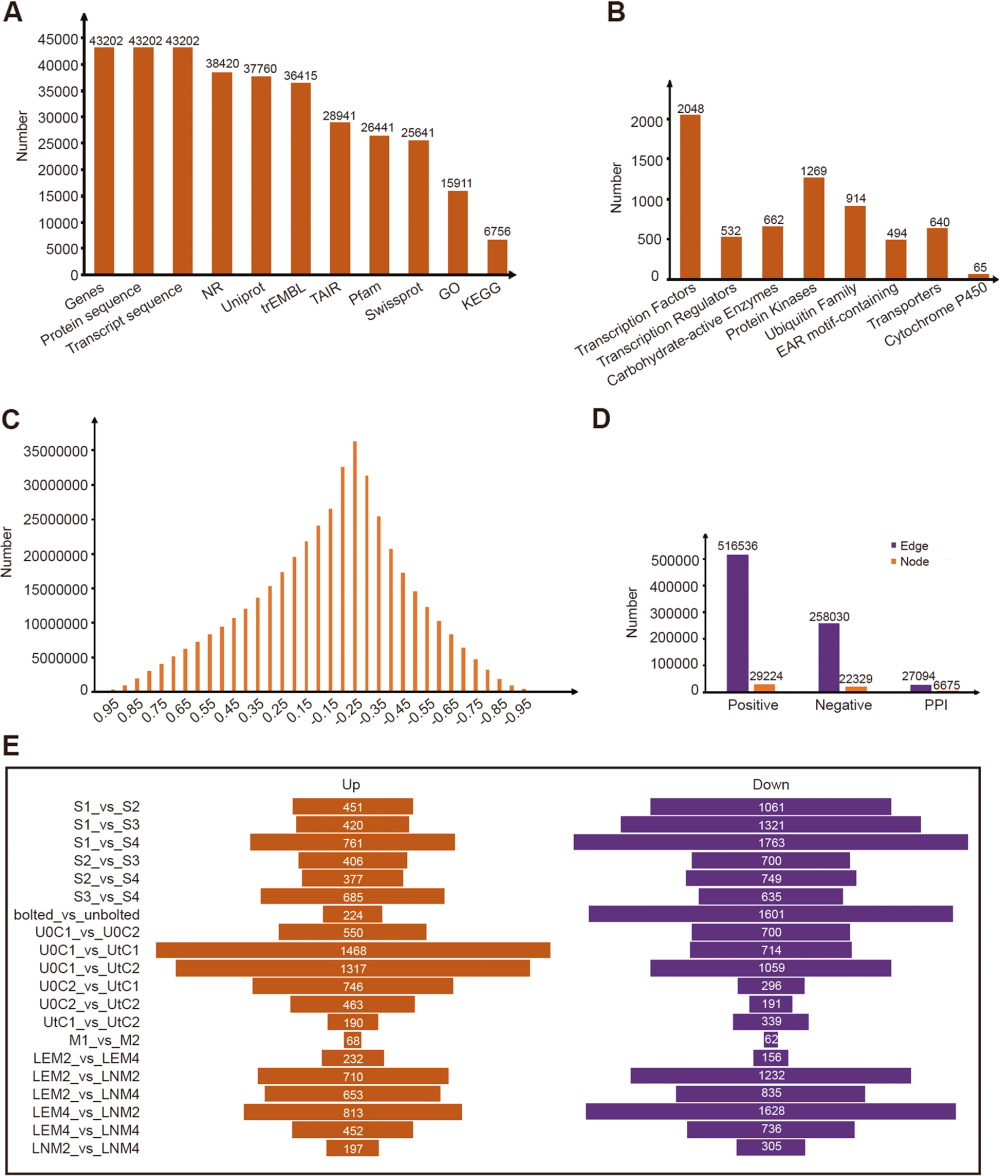
An overview of the functional annotation and network construction. **A** Number of gene sequences and annotations. **B** The distribution of gene numbers across different gene families. **C** The number of gene pairs with a changing Pearson correlation coefficient (PCC). **D** Statistics on edges and nodes in the positive and negative coexpression networks, as well as the protein‒protein interaction (PPI) network. **E** The number of DEGs in various transcriptional sample groups

First, we acquired genome information for *A. sinensis* from the cyVerse platform, encompassing a comprehensive dataset of 43,202 genes, 43,202 transcripts, and 43,202 proteins. These resources were subjected to rigorous annotation procedures by aligning the protein sequences against public databases, including NR, TAIR, UniProt, trEMBL, and SwissProt. Consequently, we successfully annotated 38,420, 28,941, 37,760, 36,415, and 25,641 genes, respectively. Furthermore, we performed Gene Ontology (GO) annotations on 15,911 genes utilizing InterProScan software [22]. To gain insight into functional pathways, we leveraged the GhostKOALA [20] online tool and mapped the KEGG annotations to 6756 genes. Finally, we conducted functional characterization of the protein domains employing PfamScan software [34] (Fig. 1A).

### Gene family classification

First, we employed iTAK software to analyze transcription factor (TF), transcription regulator (TR), and protein kinase (PK) expression in *A. sinensis*. Our analysis yielded a total of 2048 potential TFs, 532 TRs, and 1269 PKs. Next, we utilized a hidden Markov model (HMM) derived from the ubiquitin‒proteasome dataset in the iUUCD v2.0 database. This allowed us to predict 914 genes responsible for expression of ubiquitin‒proteasome components. Additionally, gene alignment against prominent databases such as PlantEAR, TransprotDB, and CAZy enabled us to identify 494 genes associated with the EAR motif-containing family, 640 genes associated with the transporter family, and 662 genes assigned to the CAZy family. Furthermore, through KEGG annotation, we successfully predicted the presence of 65 cytochrome P450 genes (Fig. 1B).

### Construction of the coexpression network

To construct a reliable coexpression network, we utilized transcriptome data consisting of 52 samples from five datasets obtained from the SRA database in the NCBI. These RNA-seq datasets were subsequently mapped to the reference genome, resulting in an overall mapping ratio exceeding 60% (Supplementary Table 1). To identify coexpressed gene pairs and evaluate the correlation between them, we first examined the distribution map of Pearson correlation coefficient (PCC) values derived from the expression profiles. The majority of the gene pairs exhibited either no correlation or a weak correlation with respect to their expression patterns (Fig. 1C). We further employed the MR (mature rank) approach to screen for gene pairs displaying strong proximity within each other's network, as determined by their respective PCC ranking values.

The positive co-expressed genes have the same expression pattern, so they may play a role in the same or similar biological processes. These similar biological processes could be evaluated by GO annotations. The higher the similarity of GO annotation of co-expressed gene pairs, the more reliable the co-expression network will be. To ensure the reliability of our constructed network, we incorporated a prior gene set based on Gene Ontology (GO) terms, specifically focusing on biological processes. GO terms associated with similar biological activities were selected, totaling 157 terms with varying gene counts ranging from 4 to 20. The next step involved determining an optimal threshold for constructing the coexpression network. We compared the area under the curve (AUC) values for different PCC values over (0.6, 0.7, 0.8, 0.9). We found that the AUC differences between PCC networks were not significant. To include more genes, we chose PCC > 0.6 as the candidate threshold (Figure S1). We also compared the area under the curve (AUC) values for different MRs with a PCC > 0.6, taking into account the overlap between the positively coexpressed genes and the previously identified GO gene sets. Through this analysis, we established network thresholds of PCC > 0.6 and MR < 50 for the positive coexpression network (Figure S2). Negative coexpression network thresholds were set at PCC < -0.5 and MR < 50. In summary, the resulting coexpression network for *A. sinensis* included approximately 774,556 coexpression gene pairs. This analysis revealed approximately 516,536 gene pairs in the positive coexpression network and 258,030 gene pairs in the negative coexpression network.

### Protein–protein interaction network

Through orthologous gene alignment with Arabidopsis PPIs sourced from TAIR (https://www.arabidopsis.org/), BAR (http://bar.utoronto.ca/welcome.htm), and BioGRID (http://thebiogrid.org/), we identified a comprehensive set of interacting proteins for *A. sinensis*. This dataset comprises 27,094 protein pairs encompassing 6675 genes (Fig. 1D).

### Network display with DEGs

To integrate gene coexpression and protein‒protein interaction (PPI) network information with gene expression data, we performed differential expression analysis on the transcriptome data, resulting in identification of differentially expressed genes (DEGs) across five sets of data. Through this process, we obtained a total of 20 distinct groups of DEGs. To visualize integration of these networks and DEGs, we created a joint display node. Within our network, upregulated DEGs are highlighted in red, and downregulated DEGs are indicated in blue. This color-coded representation allows for a clear distinction between the different expression patterns exhibited by the DEGs in the context of the network (Fig. 1E).

### Platform construction

**Fig. 2:**
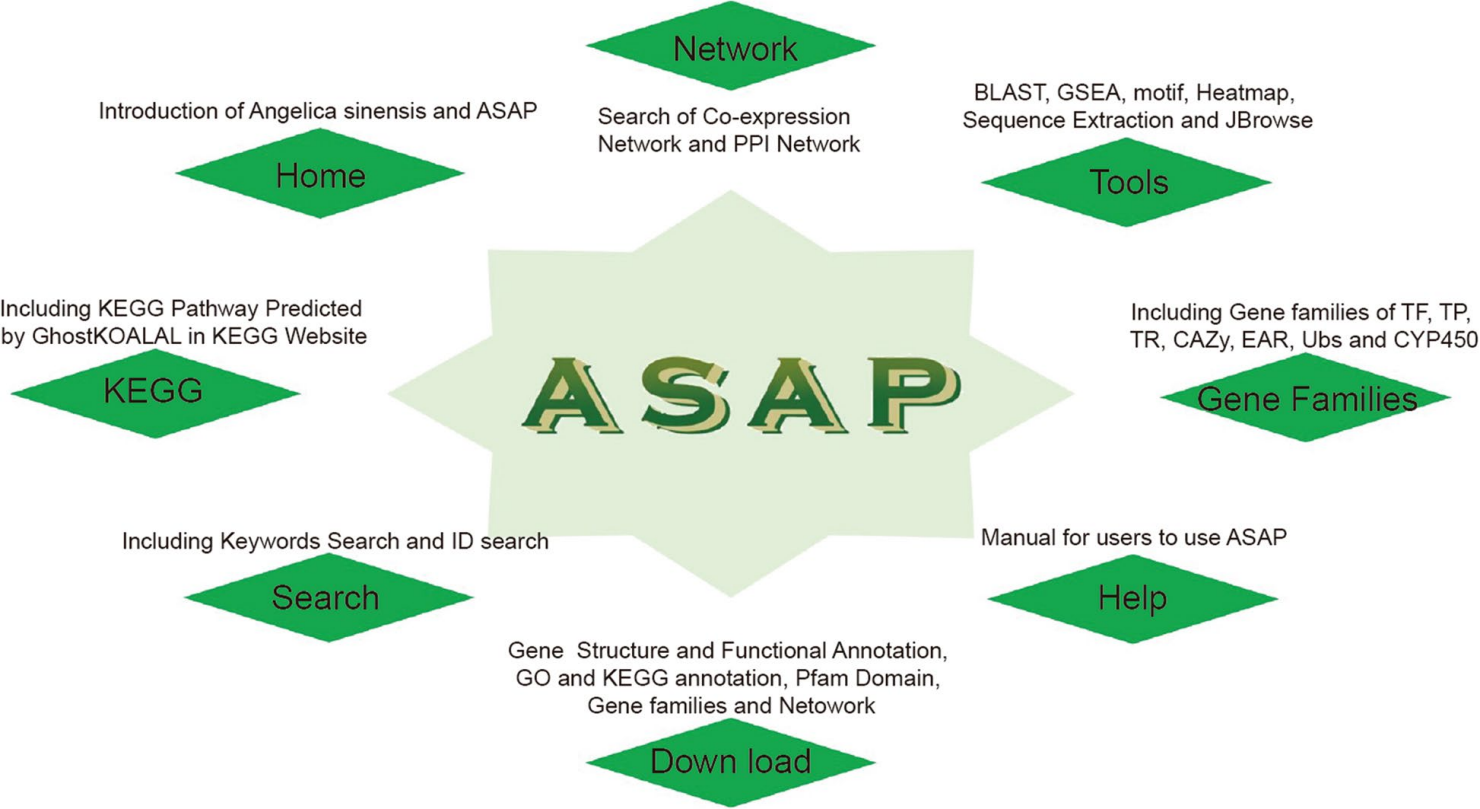
Organizational chart of the ASAP

A comprehensive platform called ASAP was developed to facilitate gene functional analysis in *A. sinensis*. ASAP consists of eight sections, Home, Network, Search, Pathway, Tools, Gene Family, Download, and Help, each designed to enhance usability and provide valuable insights for researchers (Fig. 2). The Network section offers access to both PPI and coexpression networks, enabling a deeper understanding of the intricate molecular interactions within *A. sinensis*. Our platform includes a pathway section, which primarily consists of gene annotations from the KEGG database predicted by the KOALA software. Users can access the coding genes of all key enzymes in a pathway by clicking on the corresponding pathway. The gene family section encompasses various protein families, including CYP450, TF, TR, PK, TP, ubiquitin, GAZy, and EAR motif-containing proteins. ASAP empowers researchers with a suite of tools for efficient gene functional analysis. The Search tool enables users to obtain genes of interest by utilizing keywords or precise gene, transcript, or protein accession numbers. The BLAST tool facilitates the screening of nucleic acid or protein sequences, identifying similarities within our platform. Gene Set Enrichment Analysis (GSEA) provides an inclusive approach to gene set enrichment analysis. The Extract Sequence tool allows for quick retrieval of gene sequences based on accession numbers and locations. Furthermore, the heatmap analysis tool visually presents gene expression data, facilitating interpretation of candidate gene lists. Integration of JBrowse provides an intuitive visualization of genomic and transcriptomic features, enhancing overall data exploration. The Download section provides convenient access to relevant information, ensuring easy retrieval of necessary resources. The help section offers a comprehensive user manual, guiding researchers through the platform's functionalities and optimizing their usage of ASAP. Through ASAP, researchers can perform gene functional analysis with a professional and cohesive framework, facilitating their studies in *A. sinensis*.

### Case study

1. Functional analysis of phospho-2-dehydro-3-deoxyheptonate aldolase

**Fig. 3:**
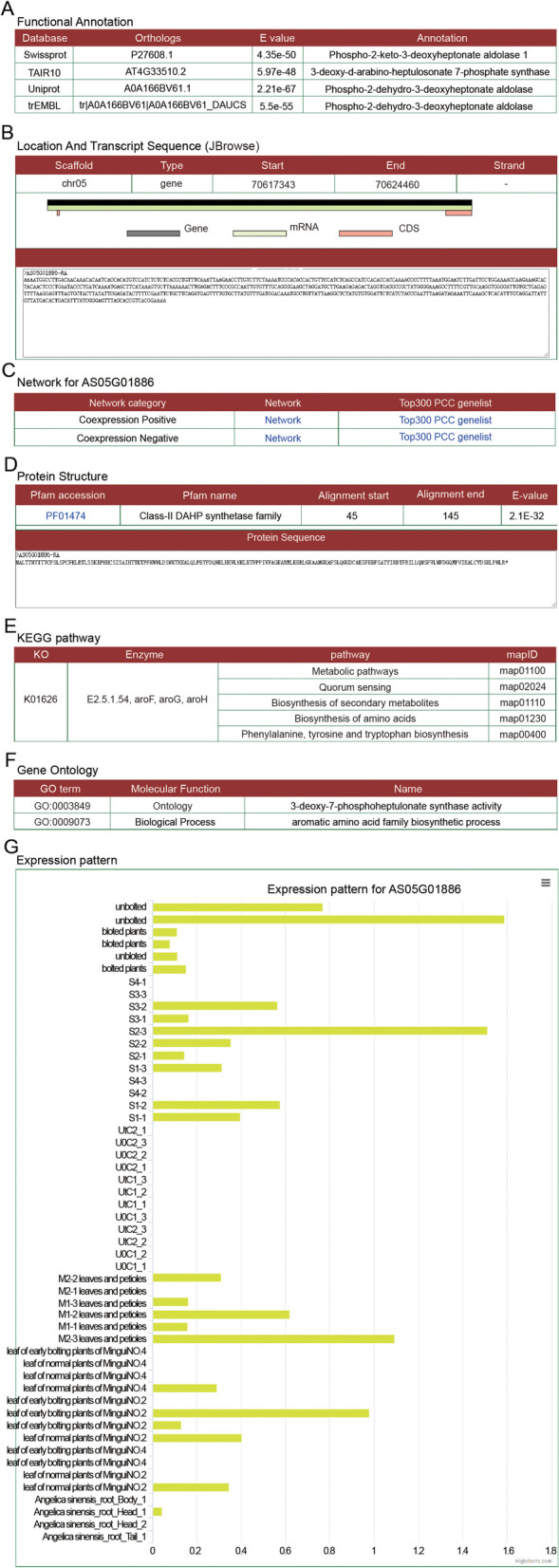
Gene detail page of the phospho-2-dehydro-3-deoxyheptonate aldolase coding gene. **A** Gene functional annotation. **B** Location and transcript sequences. **C** Network of genes encoding phospho-2-dehydro-3-deoxyheptonate aldolase. **D** Protein structure and sequence. **E** Classification of gene families. **F** KEGG annotation. **G** GO annotation. **H** Expression levels in different samples

The AS05G01886 gene in *A. sinensis* was identified as a member of the phospho-2-dehydro-3-deoxyheptonate aldolase family (Fig. 3A) and is located on chromosome 5 from 70617343 to 70624460 bp with a transcript sequence (Fig. 3B). Network links were also constructed (Fig. 3C). The Class-II DAHP synthetase family domain is located at 45 to 145 bp of the protein-coding sequence (Fig. 3D) and was identified as PF01474. GO and KEGG annotation suggested that enzyme may have a 3-deoxy-7-phosphoheptulonate synthase activity and participate in the biosynthesis of secondary metabolites (Fig. 3E, F).

**Fig. 4:**
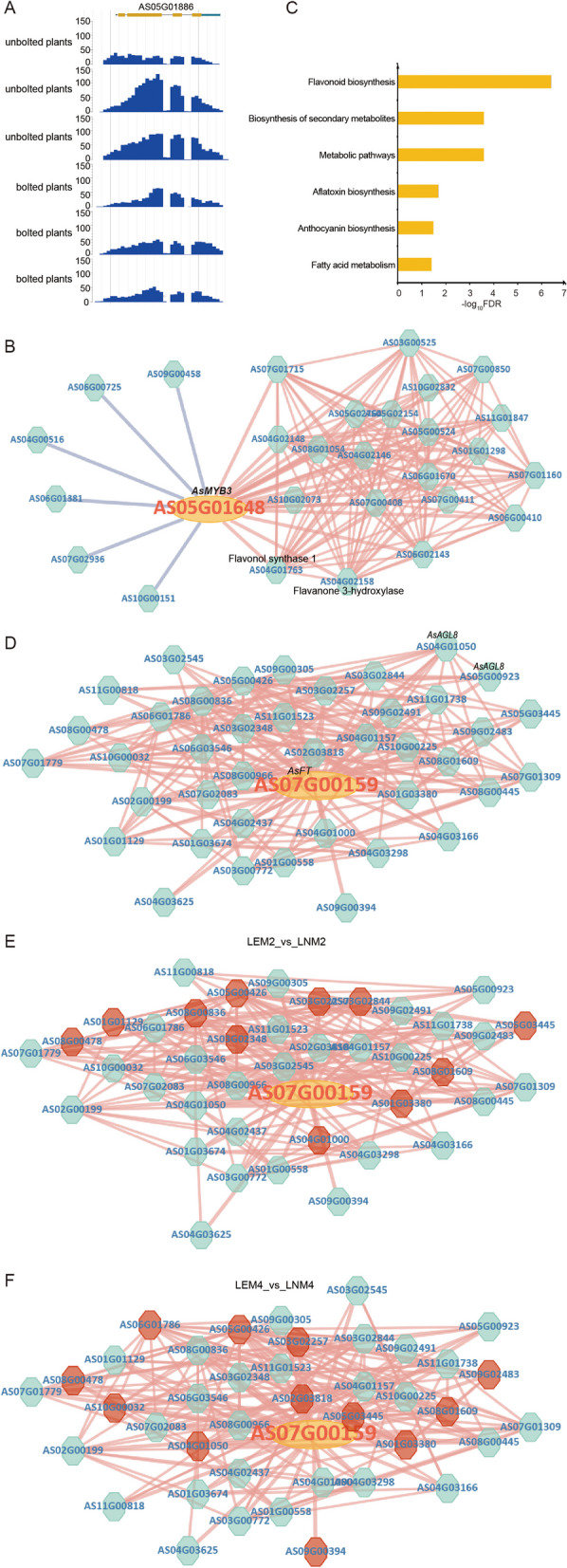
Case study for ASAP. **A** JBrowse was used to visualize expression of the gene encoding phospho-2-dehydro-3-deoxyheptonate aldolase. **B** Coexpressed genes of AsMYB3 in both the positive and negative directions. **C** Results of KEGG enrichment analysis for genes positively coexpressed with *AsMYB3*. **D** Positive coexpression network of the *AsFT* gene. **E** Comparison of coexpression networks of *AsFT* genes between early bolting plants of MinguiNO.2 and normal plants of MinguiNO.2 (LEM2_vs_LNM2). **F** Comparison of the coexpression networks of *AsTF* genes between early bolting plants of MinguiNO.4 and normal plants of MinguiNO.4 (LEM4_vs_LNM4)

Previous studies have identified a key factor, phospho-2-dehydro-3-deoxyheptonate aldolase, that may be involved in accumulation of phthalides in *A. sinensis* [13]. Through expression profiling analysis, we found that the expression level of this gene was greater in unbolted samples than in bolted samples (Fig. 3G). The display of reads mapping using JBrowse also revealed higher expression in unbolted samples than in bolted samples (Fig. 4A). Furthermore, accumulation of phthalides significantly decreases after bolting [13]. Expression of this gene showed a trend similar to that of synthesis and accumulation of active compounds. Therefore, the analysis results suggest that this factor may be involved in accumulation of phthalides.

2. Coexpression network analysis of the *AsMYB3* gene

Previous studies have suggested that *MYB3* in *Arabidopsis* regulates synthesis of anthocyanin compounds [35, 36]. We used the Blast function to compare *MYB3* in Arabidopsis with the protein sequence in the platform. Simultaneously, we filtered the results based on homology, obtained a gene with the identifier AS05G01648 and named this gene *AsMYB3*. There was a positive correlation with 21 genes and a negative correlation with 6 genes (Table S2, Fig. 4B). The key enzymes involved in the flavonoid synthesis process, namely, flavonol synthase 1 and flavanone 3-hydroxylase, also exhibited positive coexpression relationships with these genes. KEGG enrichment analysis revealed significant enrichment of pathways related to flavonoids and anthocyanins (Fig. 4C, Table S3). Therefore, *AsMYB3* may regulate involvement of these two enzymes in regulating anthocyanin production.

3. Structure and functional analysis of the *AsFT* gene

The plant *FT* gene (FLOWERING LOCUS T) is a key regulatory gene that plays an important role in the flowering process of plants [37]. The function of the *FT* gene is to regulate flowering time by interacting with other regulatory genes. FT can form complexes with genes such as CO (CONSTANS) and SOC1 (SUPPRESSOR OF OVEREXPRESSION OF CONSTANS 1), participating in initiation of flowering [37]. The AS07G00159 gene in *A. sinensis* was identified as a member of the flowering locus T (FT), located on chromosome 7 from 5234110 to 5236496 bp. Its transcript and protein sequences were also provided. Furthermore, the phosphatidylethanolamine-binding protein domain was found to be located at 52 to 161 bp in the sequence PF01161 (Figure S3).

The expression profile of this gene was significantly greater in bolted plants but not in unbolted plants in SRP232992 samples (Figure S3). In addition, *AsFT* was significantly highly expressed in the bolted samples of mingui4 and mingui2, while it was not expressed in the unbolted samples of SRP435493 (Figure S3), indicating that *AsFT* plays a positive regulatory role in controlling bolting. Furthermore, we conducted a coexpression analysis of *AsFT* with its expression profiles. Network analysis revealed 40 genes that were positively coexpressed with *AsFT* (Fig. 4D, Table S4). Among the genes that are positively coexpressed with *AsFT,* the *AGL8* gene has been shown to positively regulate flowering in many species [38, 39]. Additionally, many genes in the coexpression network were significantly upregulated in the early-flowering genotypes of mingui2 and mingui4 (Fig. 4E, F). Therefore, our analysis suggested that the *AsFT* gene plays an important role in regulating flowering, and this finding is supported by relevant studies [37].

The analysis of the examples above indicates that the platform has a certain degree of reliability and usability, offering researchers valuable assistance in exploring functional genes related to *A. sinensis*.

## Discussion

We developed a functional gene analysis platform for *A. sinensis*, aiming to provide a comprehensive resource and toolkit to help researchers gain deeper insights into functional genes and related biological processes. The coexpression network is one of the core features of our platform. By integrating a large amount of gene expression data, we constructed a coexpression network for *A. sinensis* that included genes related to *A. sinensis* and their interaction relationships. This network can assist users in discovering potential functional gene modules and regulatory pathways, thereby enhancing understanding of *A. sinensis* biological characteristics.

Our platform also provides various analysis tools for further elucidating gene functions within the coexpression network. These tools include gene set enrichment analysis, regulatory network analysis, and gene expression pattern analysis. Users can utilize these tools according to their research needs to explore the biological significance within the coexpression network. To help users access the platform effectively, we offer detailed examples of how to analyze functional genes in *ASAP*. We showcased how to filter out key genes from the coexpression network, perform gene enrichment analysis, and interpret regulatory networks. These examples not only highlight the platform's capabilities but also provide practical guidance for users in conducting their own analyses.

While our ASAP offers valuable features and tools, we are aware of potential limitations and areas for improvement. The platform currently relies on existing gene expression datasets, and the quality and coverage of the data are challenging. In the future, we plan to expand the scale and diversity of the dataset to provide more comprehensive and accurate analysis results. The analysis tools and functionalities of the platform also require further refinement and expansion. We will continue to improve the existing tools and introduce new analysis methods and algorithms to meet the evolving research needs of users. Our future plans involve continuous improvement and updates to ensure that the platform will remain in sync with the latest research advancements. We will continue to update new discoveries and technological advancements in the field of *A. sinensis* and incorporate them into the platform's features and analysis tools.

In summary, ASAP offers researchers a powerful tool for investigating functional genes and related biological processes of *A. sinensis*. By integrating coexpression networks and various analysis tools and providing detailed usage examples, we are committed to advancing *A. sinensis* research and providing valuable resources for scientists in related fields.

### Supplementary Information

**Additional file 1: Table S1.** Summary of RNA-seq datasets collected in Angelica sinensis. **Table S2.** Genes co-expressed with *AsMYB3*. **Table S3.** KEGG enrichment analysis result for *AsMYB3* co-expressed genes. **Table S4.** Genes co-expressed with *AsFT*.

**Additional file 2:** **Figure S1.** The AUC value of the co-expression network under different PCC values. **Figure S2.** The AUC value of the co-expression network under different MR values when PCC>0.6. **Figure S3.** Gene detail page of *AsFT* gene. (A) Gene functional annotation. (B) Location and transcript sequences. (C) Network of *AsFT*. (D) Protein structure and sequence. (E) Expression level in different samples.

## Data Availability

No datasets were generated or analysed during the current study.
