# Supplementary material for: ASAP: a platform for gene functional analysis in Angelica sinensis
Source: BMC Genomics. 2024 Jan 23;25:96. doi: 10.1186/s12864-024-09971-z (PMC10804808; doi:10.1186/s12864-024-09971-z)
Supplement: Supplementary file 2 — Additional file 2: Figure S1. The AUC value of the co-expression network under different PCC values. Figure S2. The AUC value of the co-expression network under different MR values when PCC>0.6. Figure S3. Gene detail page of AsFT gene. (A) Gene functional annotation. (B) Location and transcript sequences. (C) Network of AsFT. (D) Protein structure and sequence. (E) Expression level in different samples. [file 12864_2024_9971_MOESM2_ESM.docx]

***Supplementary Material***

ASAP: A platform for gene functional analysis in *Angelica sinensis*

Silan Wu^1^, Lingling Da^2^, Qiaoqiao Xiao^1*^, Qi Pan^1^, Jinqiang Zhang^1^, Jiaotong Yang^1^*

^1^Resource Institute for Chinese and Ethnic Materia Medica, Guizhou University of Traditional Chinese Medicine, Guizhou 550025, China.

^2^College of Life Science, Northwest Normal University, Lanzhou, China

***Correspondence:**

**Jiaotong Yang** ([y_jiaotong@163.com](mailto:y_jiaotong@163.com))

**Qiaoqiao Xiao** (xqqiao2021@163.com)

**Supplementary Figures**

**
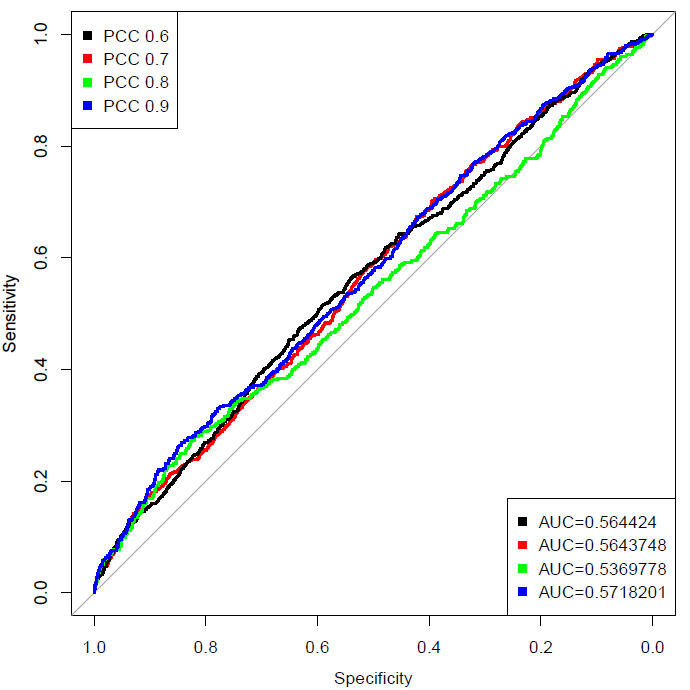
**

**Figure S1** The AUC value of the co-expression network under different PCC values.

**
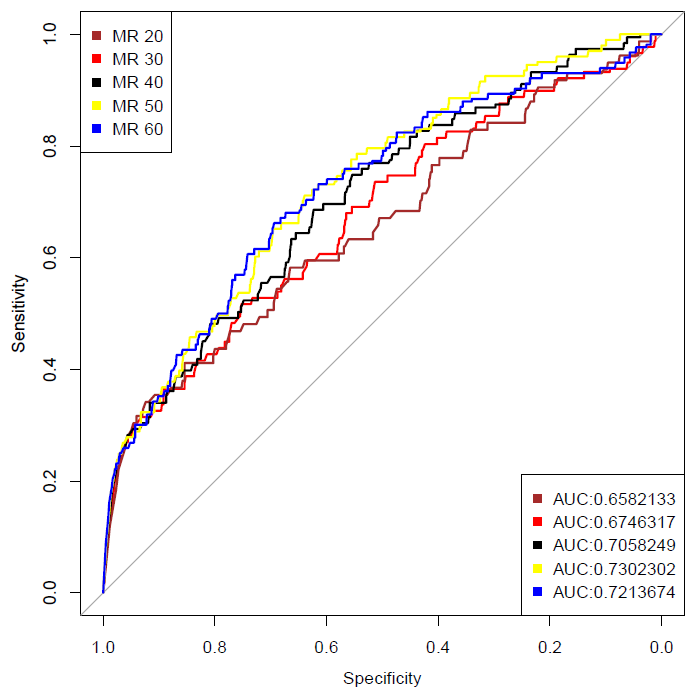
**

**Figure S2** The AUC value of the co-expression network under different MR values when PCC>0.6.


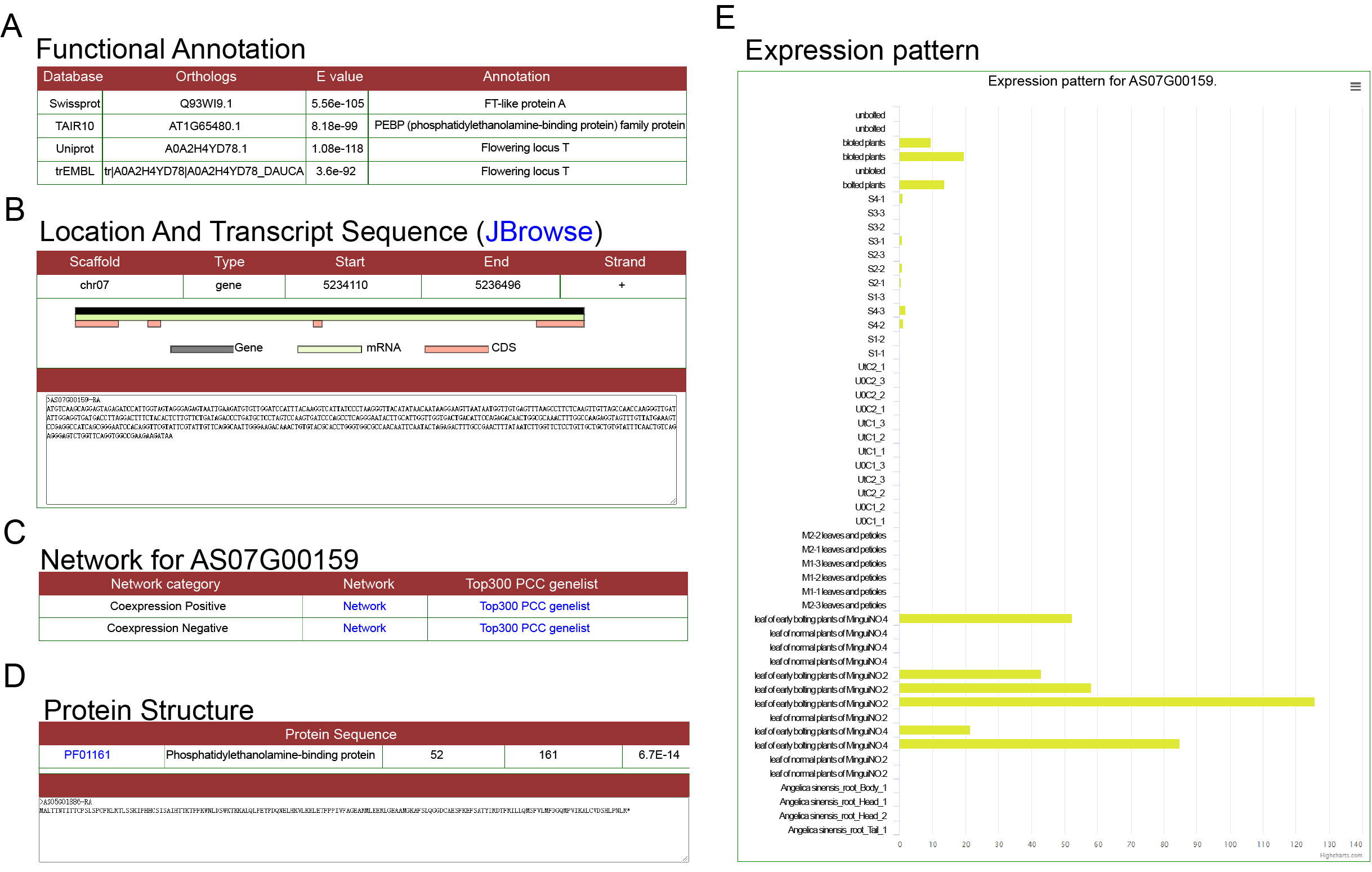


**Figure S3** Gene detail page of *AsFT* gene. (A) Gene functional annotation. (B) Location and transcript sequences. (C) Network of *AsFT*. (D) Protein structure and sequence. (E) Expression level in different samples.
